# KCNQ2-Neonatal Epileptic Encephalopathy Complicated by Ventricular Tachycardia: A Case Report

**DOI:** 10.3389/fneur.2020.00263

**Published:** 2020-04-17

**Authors:** Yuehang Geng, Xinlin Hou

**Affiliations:** Department of Pediatrics, Peking University First Hospital, Beijing, China

**Keywords:** KCNQ2, neonatal epileptic encephalopathy, ventricular tachycardia, arrhythmia, epilepsy

## Abstract

**Introduction:** Mutations in *KCNQ2* are related to a spectrum of neonatal epileptic phenotypes. Here we report a case of KCNQ2-related neonatal epileptic encephalopathy (KCNQ2-NEE) that is complicated by an incidentally found ventricular tachycardia.

**Case Presentation:** An infant boy presented with very early onset refractory focal tonic seizures and developmental delay, and was diagnosed with epilepsy. Trio-whole exome sequencing identified a previously reported *de novo* mutation in *KCNQ2* [c.794C>T; *p*. (Ala265Val)], a known pathogenic variant for KCNQ2-NEE. Interestingly, ventricular tachycardia was incidentally found on electrocardiography.

**Conclusions:** We here suggest the possibility of a potential electrophysiologic link between the two phenotypes and that they may be attributable to the same *de novo* mutation.

## Introduction

The potassium voltage-gated channel subfamily KQT member 2 gene (*KCNQ2*) is a potassium channel gene located on chromosome 20q13.3, found to be mainly expressed in the brain. Because of its important role in neuronal firing, KCNQ2-related disorders represent a broad continuum of neonatal epileptic phenotypes that range from benign familial neonatal epilepsy (BFNE) with a relatively good prognosis, to neonatal epileptic encephalopathy (NEE), which is usually associated with a poorer neurodevelopmental outcome. Other rarer phenotypes have been reported as well, including myokymia (1), benign familial infantile seizures (BFIS) (2), and infantile spasms (3).

Here we report a case of an infant boy diagnosed with neonatal epileptic encephalopathy attributable to a *de novo* mutation in *KCNQ2*, but had his case complicated by an incidentally discovered ventricular. We found this case interesting because while *KCNQ2* has not been found to be abundantly expressed in the heart, and no significant correlations have been reported between *KCNQ2* mutations and arrhythmias, it is not unreasonable to suspect that some underlying electrophysiological process may still exist to cause both epilepsies and arrhythmias in certain individuals with certain *KCNQ2* mutations.

## Case Presentation

This patient came to us at 34 days of age, but his seizures began at merely 12 h after an uneventful birth, presenting as focal tonic seizures that typically lasted from 30 s to 1 min at a time, occurring up to 30 times a day. He was administered phenobarbital (4 mg/kg.d) and levetiracetam (40 mg/kg.d), both at therapeutic dosing, at 12 days of age after CT and MRI ruled out significant intracranial hemorrhage, and video electroencephalogram (VEEG) showed possible temporal-originated focal seizures. But his seizures occurred more frequently despite upping his dosage, and he showed neurodevelopmental delay demonstrated by poor ability to follow faces or voices.

**Figure 1:**
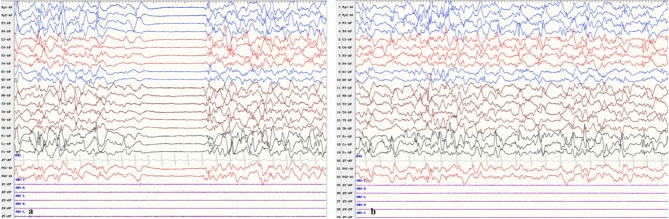
Patient EEG showing **(a)** burst suppression and **(b)** hypsarrhythmia.

**Figure 2:**
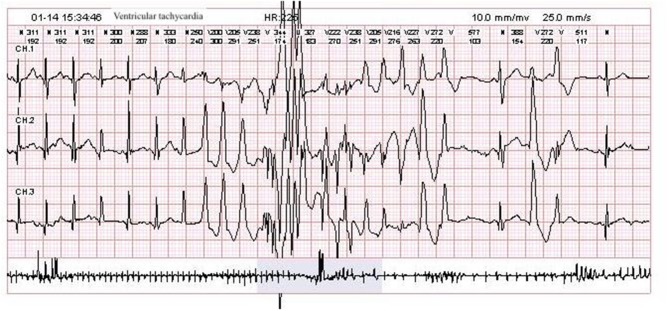
Patient Holter study showing an episode of ventricular tachycardia with varying QRS morphology.

After he was admitted, we soon ruled out infection, metabolic disturbances, and structural abnormalities of the brain via routine blood tests, blood and urine metabolic panels, cranial ultrasound, and having his previous cranial MRI consulted by our radiology team. Repeat VEEG at 35 days of age showed hypsarrhythmia and a burst suppression pattern (see Figure 1). We tapered him off phenobarbital and decided to begin a course of adrenocorticotropic hormone (ACTH) treatment in combination with topiramate (5.7 mg/kg.d), while awaiting the trio-whole exome sequencing results. Incidentally, a few probable premature atrial beats were caught on the electrocardiogram (ECG) performed as part of routine pre-ACTH evaluation, and a 24-h Holter study was immediately performed, which showed 1 premature atrial contraction, 43 premature ventricular contractions, and 5 episodes of ventricular tachycardia (VT) that lasted for 1–3 s each (HR 225–229 bpm, QTc 450 ms) all occurring within a 2-min time frame soon after a single seizure episode (see Figure 2). He was started on metoprolol (2 mg/kg.d) for rate control, and ACTH was administered for 28 days under close ECG monitoring. He showed no signs of hemodynamic instability and his heart rate gradually dropped from 160–200 to 120–140 bpm. A Holter study repeated after 3 weeks discovered no malignant arrhythmias. However, his seizures remained refractory to the multiple antiepileptic drug regimen.

Trio-whole exome sequencing results came back with a heterozygous *de novo* mutation in *KCNQ2* [c.794C>T; p. (Ala265Val)], a cytosine-to-thymine substitution of the 794th nucleotide in the coding sequence, resulting in a non-synonymous missense mutation where the 265th amino acid changes from alanine to valine. This is a known pathogenic variant of early infantile early epileptic encephalopathy with an autosomal dominant inheritance.

Mutations in *KCNQ2*, often *de novo* and rarely inherited from epileptic parents, have been reported to be associated with BFNE on the benign end, and neonatal epileptic encephalopathy (also known as KCNQ2 encephalopathy) on the severe end. We started him on oxcarbazepine (10 mg/kg.d), his seizures gradually decreased in frequency and magnitude, and he was discharged at 2 months of age.

## Discussion

KCNQ2-related neonatal epileptic encephalopathy (KCNQ2-NEE) is a rare condition starting to be increasingly recognized and reported in recent years due to its relatively severe phenotype and potentially recognizable EEG and radiological patterns (4).

In accordance with this case, KCNQ2-NEE characteristically presents with multiple daily seizures that begin during the first week of life. The seizures are mostly tonic, accompanied by motor and autonomic features, and are usually refractory to multiple antiepileptic drugs. Patients often have profound intellectual and/or psychomotor developmental impairment in later childhood, even though seizures generally cease within a few years of age. EEGs in the first week of life show a burst suppression pattern, and multifocal epileptiform activity usually develop later in the course, but may eventually return to normal after seizure control.

Our patient presented with very early-onset, intractable seizures soon after birth, not attributable to structural, electrolyte, or metabolic etiologies, and VEEG showed hypsarrhythmia with a burst suppression pattern. He had neurodevelopmental delay, and genetic test results came back with a *de novo* heterozygous c.794C>T transition in *KCNQ2*, which is a known pathogenic mutation first reported by Saitsu et al. (5) in a case of early infantile epileptic encephalopathy. The mutation resulted in an ala265-to-val (A265V) substitution, and the patient Saitsu et al. reported had very similar presentations as our patient. Therefore, we find his epileptic encephalopathy attributable to this mutation.

What caught our interest in this case was that ventricular tachycardia was found incidentally during routine evaluation. We ruled out electrolyte, metabolic, and structural abnormalities of the heart, and trio-whole exome sequencing showed no genetic variants associated with arrhythmia. Therefore, we wondered whether his arrhythmia could be another manifestation of this potassium channel gene mutation.

In 2018, Yan et al. reported a case of epilepsy due to a *de novo* mutation in KCNQ2 complicated by supraventricular tachycardia (6). However, we are the first to report a case of KCNQ2 encephalopathy complicated by ventricular tachycardia.

In recent years, numerous cases have reported epilepsy found in patients with arrythmias, suggesting that both conditions coexisting may be the result of pathogenic signaling excitability due to inherited ion channelopathy. Observations from several studies support this notion (7, 8). For example, Partemi et al. (7) conducted genetic testing on 42 individuals with epilepsy and a personal or family history of arrhythmias. They found that 24% of these individuals carried putative pathogenic mutations in genes encoding cardiac ion channels. The different phenotypes observed in ion channelopathies caused by different genetic mutations may be due to the organ-specific gene expression patterns, and inherited channelopathies involving genes regulating both cardiac and neuronal excitability may lead to a dual arrhythmia and seizure phenotype. Some of the most commonly reported genes include *SCN5A, KCNH2*, and *KCNQ1* (9, 10).

KCNQ1, as KCNQ2, is a member of the potassium voltage-gated channel subfamily. Because of its abundance of expression in the heart, mutations in *KCNQ1* are associated with congenital long QT syndrome (LQTS) and familial atrial fibrillation, accounting for approximately half of the most prevalent subtype of LQTS, LQT1 (11). Hui et al. (12) reported several electropathological mechanisms including reduced channel conductance, reduced cell surface expression, and alterations in the voltage dependence of channel activation. While *KCNQ1* is expressed more in cardiomyocytes, it has also been found to be expressed in the brain. As stated above, several cases of epilepsy associated with *KCNQ1* mutations have been reported. This may be due to the change of potassium channel properties and interference with electrophysiological functions. For *KCNQ2*, the brain is its most prominent expressing site. Zachary et al. (13) found *KCNQ2* ablation in mice led to increased neuronal excitability of neocortical layer 2/3 pyramidal neurons as well as a larger action potential amplitude, a possible mechanism of epileptogenesis. In the heart, very low levels of *KCNQ2* expression have been discovered as well (14), in theory, if the mutation had a functional impact on action potential in the cardiomyocyte, it is possible for patients carrying certain mutations in *KCNQ2* to present with both epilepsy and arrhythmia. But currently no study has found evidence of KCNQ2 impacting cardiac action potentials.

Another possible explanation for the occurrence of ventricular tachycardia in patients with epilepsy, according to recent literature, is that through certain mechanisms yet unknown, epilepsy has caused a “pro-arrhythmic” state. For example, animal studies have shown that seizure activity alters the expression of ion channels in the heart, leading to abnormal ventricular repolarization and greater susceptibility to ventricular arrythmias and QTc prolongation (15).

Although by this point neither our case nor the cases referred to above can demonstrate a definitive link between epilepsy and arrhythmia in patients with ion channelopathies, they provide us with more potential genetic variants to look for in patients with either condition, and could possibly help decrease their risk of sudden unexpected death either due to epilepsy or cardiac events.

## Ethics Statement

We have obtained a written consent from the patient's parents for the publication of this case report.

## Author Contributions

YG wrote the paper under advisement of XH.

## Conflict of Interest

The authors declare that the research was conducted in the absence of any commercial or financial relationships that could be construed as a potential conflict of interest.
